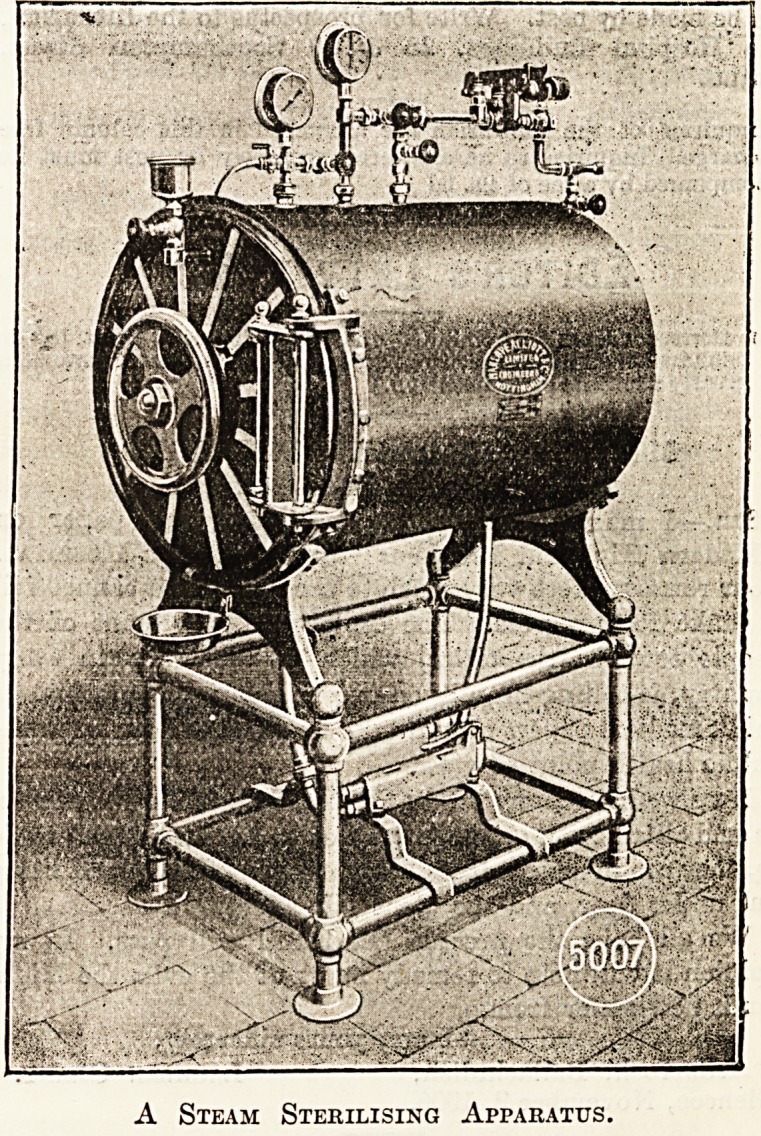# Structural and Practical Departments

**Published:** 1906-11-10

**Authors:** 


### STRUCTURAL AND PRACTICAL DEPARTMENTS.

### A STEAM STERILISING APPARATUS FOR USE IN OPERATING THEATRES.

Messrs. Manlove Alliott and Co., of Nottingham, who are so well known in connection with their improved Lyons disinfector, laundry machinery, refuse destructors, and other apparatus, have introduced a steam sterilising apparatus for use in operating theatres, which they claim

A Steam Sterilising Apparatus.

can be handled quite easily by nurses. It is practically a *replica* on a small scale of their disinfector. It consists of a cylinder, copper-faced on the inside, and with copper trays for holding the instruments, bandages, etc., to be sterilised, with ledges for the trays to rest upon. The cylinder has a jacket outside of it, practically another cylinder, enclosing an annular space, into which steam or liquids may be introduced for the purpose of heating, etc. The cylinder is closed by a door, carefully fitted, so as to swing easily on its hinges, and to close steam-tight without the use of asbestos, rubber, etc. The apparatus is fitted with the vaccum arrangement that has been so long in use with the disinfector, and is arranged to be heated by the admission of steam from the boiler service to the jacket, or the jacket itself may be used as a boiler, any kind of sterilising liquid being employed in it, and the liquid in the jacket may be heated either by a steam coil supplied from the boiler service, or by an ordinary ring burner underneath. In sterilising bandages, dressings, etc., the air is first exhausted from the sterilising chamber to a certain extent, the remaining air is then heated either from the jacket or directly by steam passed into the chamber, and the fabrics are dried and made ready for immediate use, after the sterilising is complete, by a second use of the vacuum apparatus, the steam and moisture being all withdrawn. The apparatus is made in three sizes, respectively, 12 in., 18 in., and 24 in. in diameter, the lengths being 24 in., 30 in., and 36 in. One form of it is shown in the illustration.

## Figures and Tables

**Figure f1:**